# scSNPdemux: a sensitive demultiplexing pipeline using single nucleotide polymorphisms for improved pooled single-cell RNA sequencing analysis

**DOI:** 10.1186/s12859-023-05440-8

**Published:** 2023-08-31

**Authors:** John K. L. Wong, Lena Jassowicz, Christel Herold-Mende, Martina Seiffert, Jan-Philipp Mallm, Peter Lichter, Marc Zapatka

**Affiliations:** 1 Division of Molecular Genetics, German Cancer Research Center, Heidelberg, Germany; 2 Division of Experimental Neurosurgery, Department of Neurosurgery, Heidelberg University Hospital, Heidelberg, Germany; 3 Single-Cell Open Lab, German Cancer Research Center, Heidelberg, Germany

**Keywords:** Single-cell, Sample pooling, Sample demultiplexing, Single nucleotide polymorphisms

## Abstract

**Background:**

Here we present scSNPdemux, a sample demultiplexing pipeline for single-cell RNA sequencing data using natural genetic variations in humans. The pipeline requires alignment files from Cell Ranger (10× Genomics), a population SNP database and genotyped single nucleotide polymorphisms (SNPs) per sample. The tool works on sparse genotyping data in VCF format for sample identification.

**Results:**

The pipeline was tested on both single-cell and single-nuclei based RNA sequencing datasets and showed superior demultiplexing performance over the lipid-based CellPlex and Multi-seq sample multiplexing technique which incurs additional single cell library preparation steps. Specifically, our pipeline demonstrated superior sensitivity and specificity in cell-identity assignment over CellPlex, especially on immune cell types with low RNA content.

**Conclusions:**

We designed a streamlined pipeline for single-cell sample demultiplexing, aiming to overcome common problems in multiplexing samples using single cell libraries which might affect data quality and can be costly.

**Supplementary Information:**

The online version contains supplementary material available at 10.1186/s12859-023-05440-8.

## Introduction

Single cell sequencing is a powerful method for studying tissue heterogeneity and biological replicates are analysed to gain statistical power for such experiments. A major hurdle of expanding the number of samples in experiments is the cost of single-cell library preparation. Pooling multiple specimens is one way to reduce costs. Wet-lab sample multiplexing techniques, such as lipid-based [1] or hashtag antibody-based methods were developed to pool multiple specimens into one library. Meanwhile, attempts were made to extract genotype information from 5′ or 3′ single cell RNA sequencing data. For example, cellsnp-lite allows users to extract germline single nucleotide polymorphism (SNP) information from individuals sequenced by 10× Genomics protocols [2]. Therefore single-cell sequencing provides SNP information that permits in-silico sample separation when multiplexed libraries are prepared from different individuals [3]. However, SNP-based approaches do not support the demultiplexing of multiple specimens from the same individual due to the shared SNP information. The use of in-silico demultiplexing approaches over costly wet-lab procedures have raised interest in the scientific community, but questions remained on its performance [4]. Meanwhile, input processing of each step required for SNP demultiplexing could be time consuming and error prone.

Here, we present a streamlined pipeline that takes outputs of Cell Ranger from 10× Genomics and genotyping data from either SNP arrays or high-throughput sequencing to retrieve the correct identity of single cells. Our pipeline provides high sensitivity and doublets removal functionality in one command. We observed superior sample demultiplexing performance of scSNPdemux compared to CellPlex by applying it on two datasets, breast cancer and non-small cell lung cancer (NSCLC) samples, respectively. Compared to Multi-seq the assignment was consistent for demultiplexed cells while scSNPdemux enabled more than doubling the number of demultiplexed cells. The SNP-based demultiplexing approach allows improved detection of doublets within multiplexed datasets, making estimation of doublet populations and characterization possible and thereby leading to improved data quality.

## Implementation

In short, scSNPdemux is an R package that combines multiple tools to identify informative SNPs on single cell level, to demultiplex the mixture to assign individual cells to samples, and to call potential doublets. Identities of cells are informed by SNP contents per cell-barcode, where cellsnp-lite calls SNPs separately on each barcode. Cellsnp-lite was selected for extracting SNP information (base specific read depths) because of its speed and memory efficiency [2]. Cell barcodes derived from the same individual are thereby identified by their genetic distance inferred by a collection of SNPs. Cells defined by a unique cell barcode that show multiple SNP genotypes from different individuals and therefore are thought to have originated from individuals with different germlines are reported as “doublets”. This doublet detection and the hash based CellPlex approach however are limited to cell doublets originating from more than one individual.

ScSNPdemux input requires a BAM file of single cell RNA sequencing reads aligned by Cell Ranger, a gene expression count matrix from Cell Ranger, population SNP information (e.g. 1000 genomes phase 3) and genotyped SNPs from the multiplexed samples in VCF format, which are mapped to human genome reference GRCh38. The SNP genotype information for the multiplexed samples can be prepared from high-throughput DNA sequencing or SNP arrays. Initially, SNPs are called from the aligned reads on sites with population frequency of > 5% within the 1000 genomes phase 3 dataset using cellsnp-lite. To include more information for improved sample demultiplexing, sample genotypes are merged from the outputs of cellsnp-lite and the input genotypes using bcftools [5]. By combining these SNP genotypes, we maximize the sensitivity of the demultiplexing tool vireo [3], especially when the amount of SNPs identified by the genotyping array is scarce.

As a result, the pipeline reports the most likely sample identity, or whether the cell barcode could be linked to a cell doublet (as tab-delimited file). The output file can thus be loaded into a Seurat object as metadata, or assigned to the sample identity field for any downstream analysis.

The demultiplexing workflow is available on github (https://github.com/wkljohn/scSNPdemux). To benchmark the performance of our pipeline, we retrieved a dataset of 7 NSCLC from 10× Genomics which are multiplexed by 10× CellPlex library (40 k Mixture of NSCLC DTCs from 7 donors, 3' HT v3.1, (https://www.10xgenomics.com/resources/datasets/40-k-mixture-of-nsclc-dt-cs-from-7-donors-3-ht-v-3-1-3-1-high-6-1-0). InferCNV [6] was used to call copy number changes. By assuming all cancer cells in these datasets have copy number aberrations, we were able to define cancer cell clusters when more than 8 long segments of genomic regions spanning more than 10 Mb are observed on 80% of cells.

## Results

**Fig. 1:**
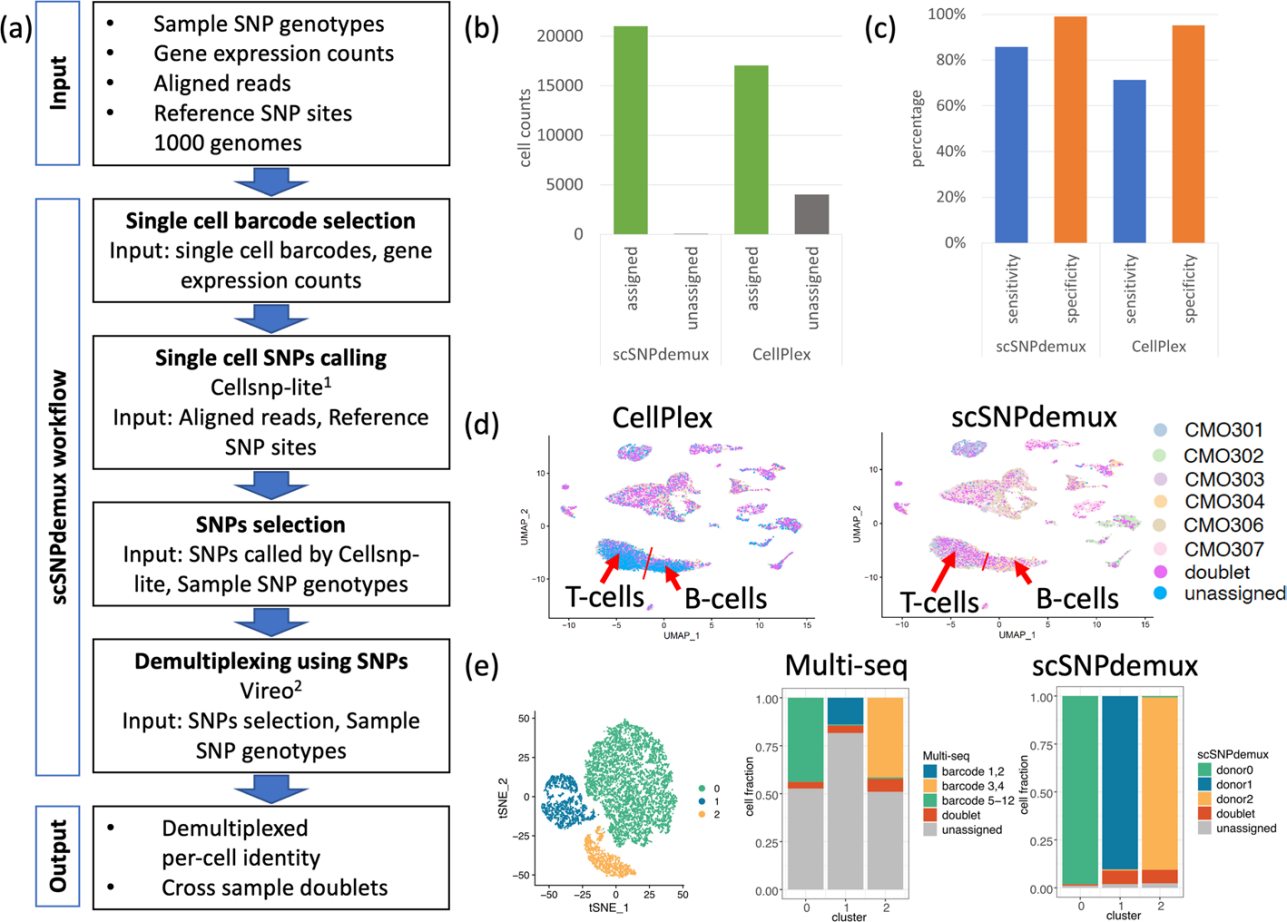
**a** Description of the scSNPdemux workflow. **b** A barplot summarizing the number of assigned cells independently by CellPlex and scSNPdemux. **c** The sensitivity and specificity estimation by copy number defined tumour clusters, assuming tumour cells from different patients share a similar copy number profile and form distinct clusters on transcriptome level. **d** The comparison between CellPlex and scSNPdemux by overlaying the demultiplexed cell labels onto the UMAP. **e** Comparison of demultiplexing a mixture of Jurkat, HEK293 and HMEC cells by Multi-seq and scSNPdemux. T-SNE map showing three cell types and barplots presenting the fraction of cells assigned. (1) Huang and Huang [2], (2) Huang et al. [3]

We first applied scSNPdemux (Fig. 1a) to demultiplex the 40 k Mixture of NSCLC DTCs (see above) from 7 donors. ScSNPdemux assigned sample identities to 19,186 of 21,071 cells and flagged 1822 cells as doublets using SNPs genotyped per cell barcodes. Comparing to the CellPlex lipid-based sample demultiplexing, scSNPdemux was able to assign the sample identity to additional 19.1% of the cells (4021 of 21,071 cells) (Fig. 1b,c).

To assess the accuracy and performance of the two demultiplexing methods, we analysed the cell identity allocation per expression-based tumour cell clusters. Tumour-cell clusters were identified by a high degree of copy number changes in contrast to copy number neutral profiles found in normal cells. Tumour cells from different patients typically form independent clusters and the assumption was verified by the homogeneous copy number profiles observed in each tumour cluster (Additional file 1: Fig. S1). This characteristic was employed to assess the accuracy of the two sample demultiplexing methods. The comparison revealed scSNPdemux outperforms CellPlex in both sensitivity (86% vs. 71%) and specificity (99% vs. 95%) (Fig. 1c). The presence of copy number variations in the tumour cells might impact the demultiplexing process. However, scSNPdemux demonstrated a higher number of assigned cells compared to CellPlex across all cell types, we do not anticipate an overestimation of specificity (see Additional file 1: Table S1 for number of assigned cells and identified duplicates across celltypes).

To functionally interpret the cells that were in addition demultiplexed by scSNPdemux we performed cell type annotation by the Azimuth algorithm [7] (Additional file 1: Fig. S2). Of those cells, 3257 of 4021 were annotated as CD3 expressing T-cells or CD19 expressing B-cells. ScSNPdemux achieved 91% sensitivity on cells in the T-cell cluster (6355 of 6999 cells in the cluster assigned), compared to 62% sensitivity using CellPlex (4319 of 6999 cells assigned). Furthermore, our pipeline achieved 92% sensitivity on B-cells clusters (3247 of 3521 cells in the cluster assigned), compared to 59% using CellPlex (2060 of 3521 cells in the cluster assigned). The mean read counts for all clusters is 17,563, T-cell clusters is 5569, and B-cell clusters is 6793. Accordingly, the analysis of immune cells that cannot be demultiplexed by CellPlex due to low overall transcriptional activity, showed the biggest improvements when applying scSNPdemux (Fig. 1d, Additional file 1: Figs. S5, S6).

Application on a second dataset of three patients with breast cancer demonstrated that scSNPdemux can also be applied to single nucleus RNA sequencing data with genotypes profiled by SNP arrays. Here, we were able to accurately resolve the sample identities of 9329 cells and identified 1020 cell doublets based on sample genotype information extracted from Illumina Oncoarray-500 K, which provides information on a smaller number of SNPs. Analysis of demultiplexing results on CNV defined tumour clusters revealed a sensitivity and specificity of scSNPdemux of 100% and 99.7%, respectively (Additional file 1: Fig. S4). This analysis demonstrated the potent ability of scSNPdemux to resolve sample identities using sparse genotyping data.

In addition, we applied scSNPdemux to a lipid-based Multi-seq barcoding data set multiplexing three human cell lines Jurkat, HEK293, HMEC [1]. We processed the nuclear single RNA sequencing from SRR8890625, SRR8890636 and SRR8890648 with Seurat (Seurat_4.9.9.9044) using default settings and compared the assignment to the three cell clusters (Fig. 1e, Additional file 1: Fig. S7) by Multi-seq and scSNPdemux. Interestingly, when analysing the lipid-based Multi-seq data, we observed that Multi-seq it failed to assign a significant fraction of the 12,665 cells containing more than 200 UMI barcodes (57.4%, 7272 cells) compared to scSNPdemux (1.4%, 180 cells) (Fig. 1e). Analysing the cells not assigned to any cluster, we showed that especially cells with a lower UMI count were missing in the scSNPdemux assignments. In the Multi-seq approach, many cells with a high UMI count profile could not be assigned (Additional file 1: Fig. S8). Despite this discrepancy in assignment rates, the overlap of cell assignments to the three clusters between the two approaches was remarkably high (99.85%), with only seven out of 4816 cells showing different cluster assignments (Additional file 1: Table S3). This indicates a strong overall agreement in the cell assignments, with scSNPdemux assigning a substantially higher number of cells.

## Conclusion

Our pipeline scSNPdemux utilizes natural genetic variations in humans from sparse to dense SNP genotyping data for demultiplexing pooled single-cell RNA sequencing data at high sensitivity and specificity. Using a set of NSCLC sc data obtained by 10× Genomics, scSNPdemux identified substantially more immune-cells over CellPlex. Our pipeline offers better demultiplexing results for cells with low read counts and is therefore especially helpful when studying the immune cell component using single-cell RNA sequencing. Our breast cancer dataset also demonstrated that SNP-array data is sufficient for accurate patient ID demultiplexing from single nuclei. Comparing the performance with Multi-seq also demonstrated and outstanding improvement of cell assignments. Library preparation costs can be reduced while having the benefit of an accurate sample demultiplexing and doublet detection if samples from different individuals are multiplexed. The pipeline is compatible with standard bioinformatics workflows of single cell sequencing analysis and provides a cost-effective way to increase the number of biological replicates while maintaining high data quality.

### Availability and requirements

- Project name: scSNPdemux.
- Project home page: https://github.com/wkljohn/scSNPdemux.
- Operating systems: Linux.
- Programming language: Shell script and R.
- Other requirements: R 4.0 or higher.
- License: GNU General Public License v3.0.
- Any restrictions to use by non-academics: license needed for commercial use.

### Supplementary Information

**Additional file 1:** Supplementary Figures and Tables.

## Data Availability

The scSNPdemux pipeline is available on https://github.com/wkljohn/scSNPdemux. The dataset of 7 NSCLC from 10X Genomics multiplexed by 10X CellPlex analysed during the current study is available at 10X Genomics (40k Mixture of NSCLC DTCs from 7 donors, 3' HT v3.1, https://www.10xgenomics.com/resources/datasets/40-k-mixture-of-nsclc-dt-cs-from-7-donors-3-ht-v-3-1-3-1-high-6-1-0).

## Abbreviations

CNV: Copy number variation
NSCLC: Non-small cell lung cancer
RNA: Ribonucleic acid
SNP: Single nucleotide polymorphisms
