# Supplementary material for: scSNPdemux: a sensitive demultiplexing pipeline using single nucleotide polymorphisms for improved pooled single-cell RNA sequencing analysis
Source: BMC Bioinformatics. 2023 Aug 31;24:326. doi: 10.1186/s12859-023-05440-8 (PMC10469441; doi:10.1186/s12859-023-05440-8)
Supplement: Supplementary file 1 — Additional file 1: Supplementary Figures and Tables. [file 12859_2023_5440_MOESM1_ESM.docx]

## Supplementary Information

Contents

[Supplementary Information 1](#_Toc141097830)

[Supplementary figure 1: UMAP plots and copy number variants in 7 lung cancer samples(NSCLC) profiled by scRNASeq 2](#_Toc141097831)

[Supplementary figure 2: Azimuth annotation of UMAP clusters from 7 NSCLC 4](#_Toc141097832)

[Supplementary figure 3: Cell assignment summary 5](#_Toc141097833)

[Supplementary figure 4: UMAP plots and copy number plots for 3 breast cancer samples profiled by snRNASeq 6](#_Toc141097834)

[Supplementary figure 5: UMAP plots on T-Cell subclustering. 8](#_Toc141097835)

[Supplementary figure 6: UMAP feature plots on subcluster markers contrasting different sample identity allocation strategies. 9](#_Toc141097836)

[Supplementary figure 7: t-SNE plots comparing Multi-seq and scSNPdemux assignments. 11](#_Toc141097837)

[Supplementary figure 8: UMI counts per cell across demultiplexing results 12](#_Toc141097838)

[Supplementary information 13](#_Toc141097839)

[Supplementary Table 1 13](#_Toc141097840)

[Supplementary Table 2 Assigments of the multiplexed Jurkat, HEK293 and HMEC cells from McGinnis et al. 2019 14](#_Toc141097841)

[Supplementary Table 3 Overview of cells assigned by Multi-seq and scSNPdemux 15](#_Toc141097842)

[Subclustering comparison between all identified cells and cells only identified by scSNPdemux 16](#_Toc141097843)

### Supplementary figure 1: UMAP plots and copy number variants in 7 lung cancer samples(NSCLC) profiled by scRNASeq

(a) UMAP clusters generated by Seurat using single cell expression data from 7 NSCLC. Cells were grouped into 24 clusters. (b) Copy number profiles are plotted per UMAP cluster. Copy number analysis was performed by inferCNV, the plot was split into upper and lower panels where the upper panel shows UMAP clusters from non-tumour cells and the lower panel shows copy number changes in tumour clusters. Copy number gains are shown in red and copy number loss are shown in blue.

(a)

(b)

Cluster

20

11

10

6

5

15

16

9

21

4

23

22

14

8

12

0

1

2

3

7

13

17

18

19


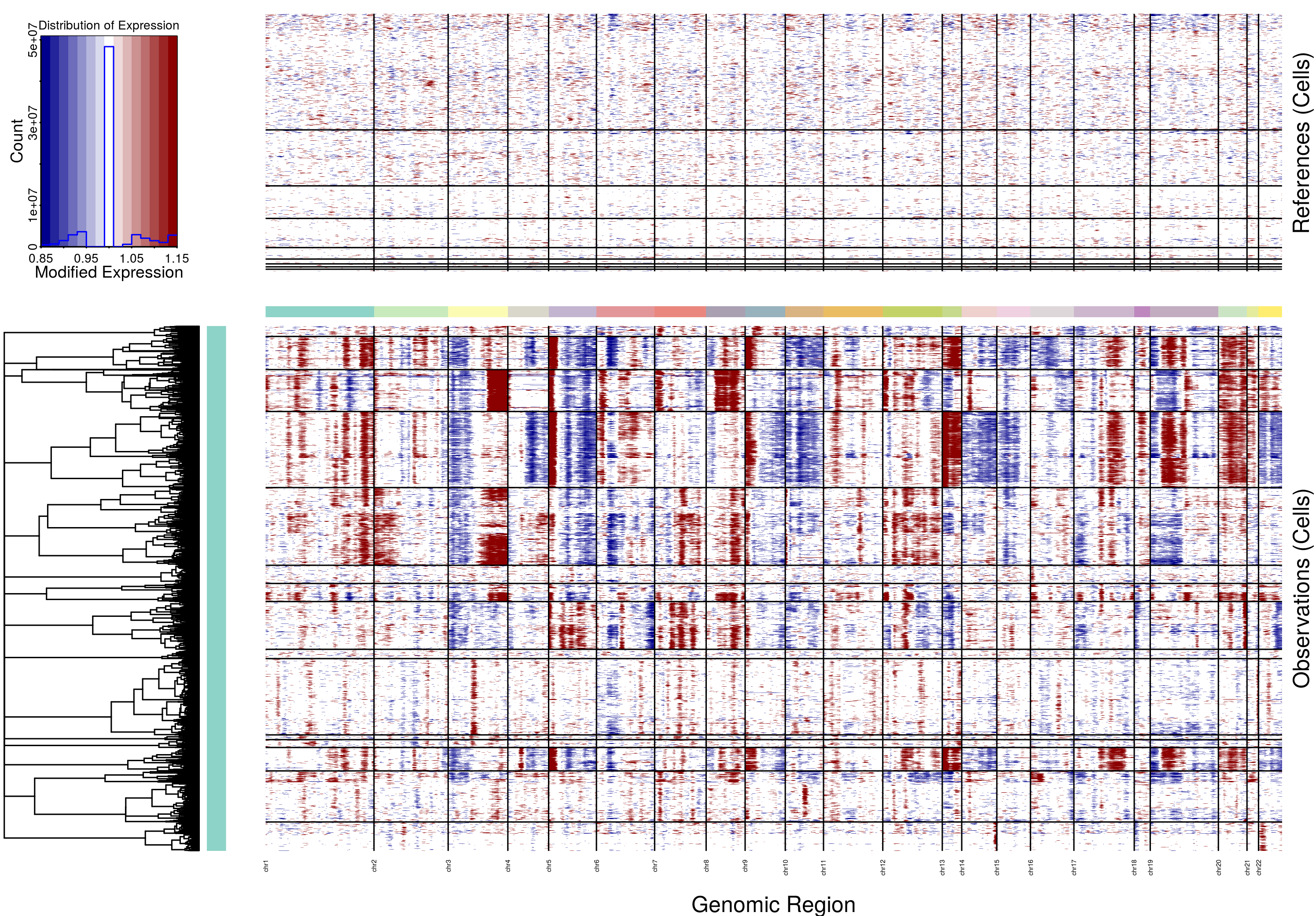


### **Supplementary figure 2: Azimuth annotation of UMAP clusters from 7 NSCLC**

Cell type annotation by Azimuth using the reference “Human – Lung V2(HCLA)”. Cell clusters annotated as immune cells and non-tumour cell types were defined by missing copy number aberrations defined by inferCNV analysis across 7 NSCLCs and used for downstream analysis.

### Supplementary figure 3: Cell assignment summary

A summary table showing summary statistics of cells assigned by CellPlex and scSNPdemux in 3 categories.

|  | Cellplex | scSNPdemux |
| --- | --- | --- |
| assigned | 14003 | 19186 |
| doublets | 3028 | 1822 |
| unassigned | 4040 | 63 |

### Supplementary figure 4: UMAP plots and copy number plots for 3 breast cancer samples profiled by snRNASeq

(a) UMAP clusters generated by Seurat using single cell expression data from 3 breast cancer samples Cells were grouped into 24 clusters, where the clusters groupings are used for the subsequent copy number variation analysis. (b) UMAP with sample identity annotation using the sample demultiplexing results from scSNPdemux, (c) Copy number profiles inferred by infer CNV plotted per UMAP cluster. The upper panel shows copy number profiles from non-tumour clusters and the lower panels shows copy number from tumour clusters of the 3 breast cancer samples. Copy number gains are shown in red and copy number loss are shown in blue.

(a)

(b)


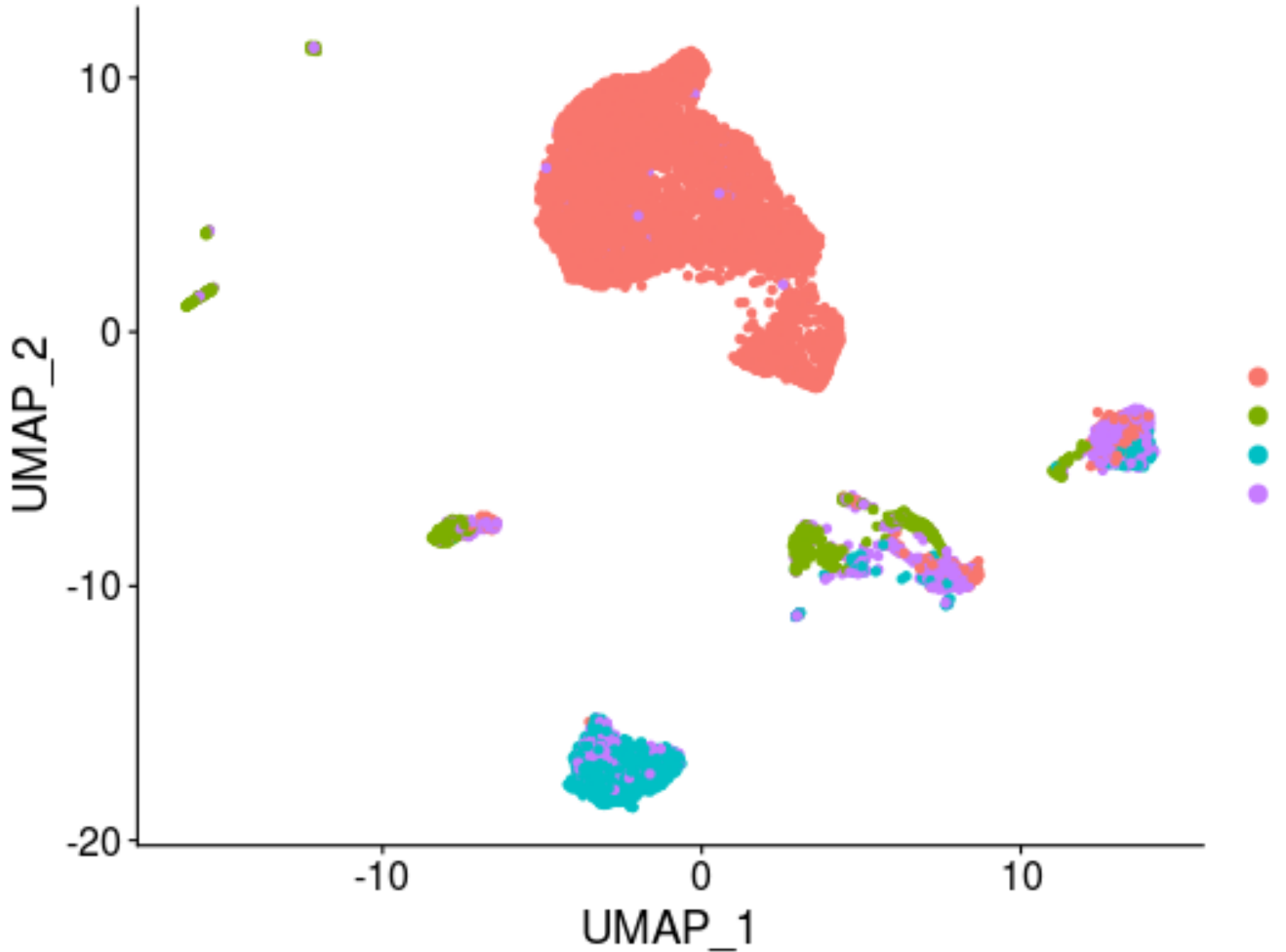


donor1

donor2

donor3

doublets

(c)

Cluster

donor1

donor2

donor3

doublets

19

11

17

5

9

2

0

3

1

16

4

7

21

10

12

20

18

15

8

14

6

13


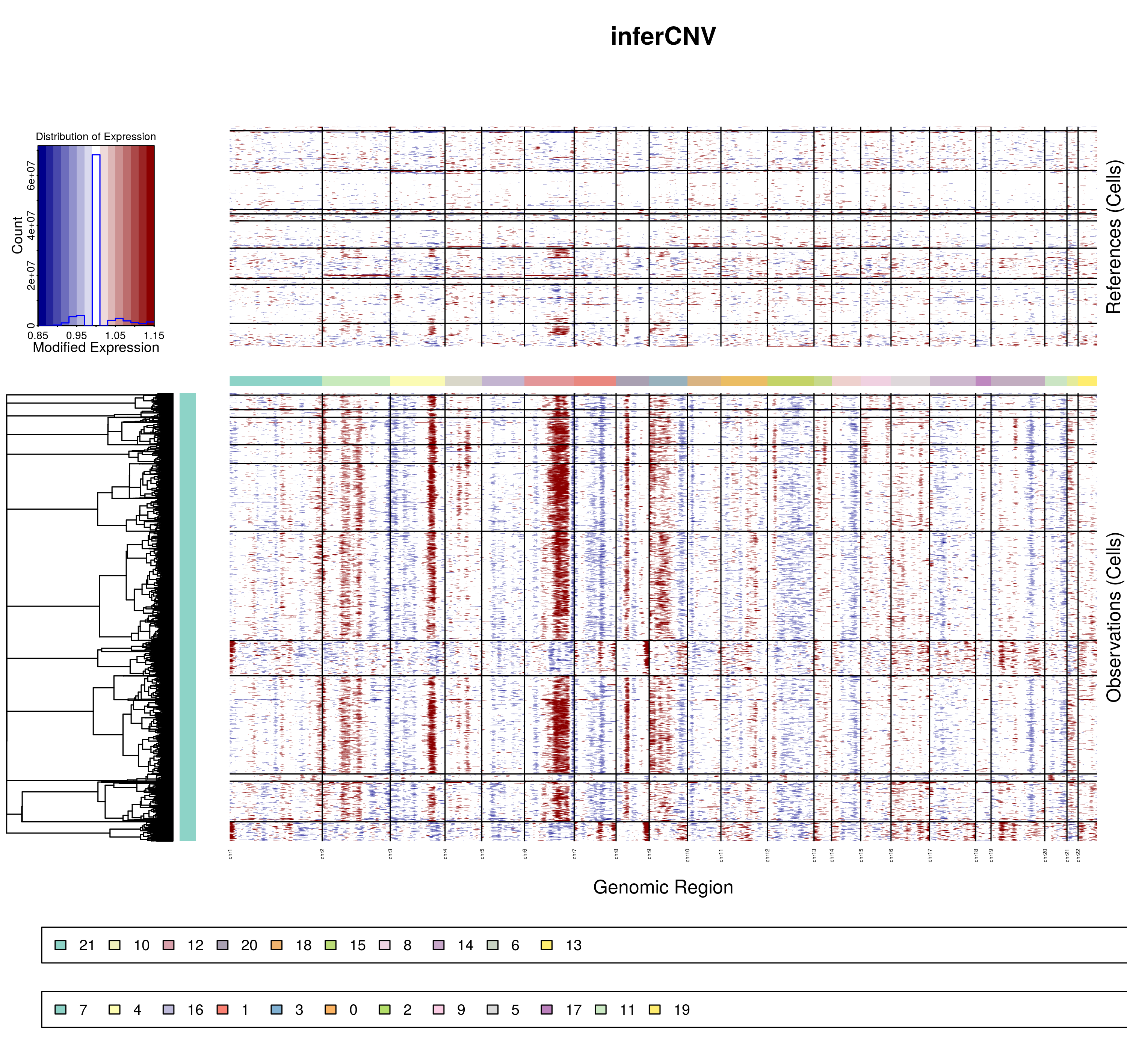


### Supplementary figure 5: UMAP plots on B/T-Cell subclustering.

Subclustering analysis were performed separately on (a) B/T-Cells with identity assigned by scSNPdemux or CellPlex, (b) B/T-Cells with identity only assigned by scSNPdemux

(a)

(b) Cell cluster labels from (a) transferred to a newly computed UMAP including only cells with sample identity assigned exclusively by scSNPdemux using the same colour scheme as in supplementary figure 5a for transferred clusters.

### Supplementary figure 6: UMAP feature plots on subcluster markers contrasting different sample identity allocation strategies.

Cluster-defining markers from the full population of T-Cells were used to perform in-silico staining of clusters of cells only identified by scSNPdemux . In the sections from (a) to (d) below, the plot on the left indicates the UMAP from full population of T-Cells where the plot on the right indicates the UMAP from the cell population identified only by scSNPdemux.

1. CD4

1. CD8

1. CXCL13

1. MS4A1

1. PDCH9

### Supplementary figure 7: t-SNE plots comparing Multi-seq and scSNPdemux assignments.

t-SNE plots depicting demultiplexed nuclear single RNA sequencing data from Jurakt, HEK293 and HMEC cells (SRR8890625, SRR8890636 and SRR8890648) a) presenting clustering of cells using Seurat b) presenting barcode assignments from McGinnis et al. Nat Methods 2019 c) presenting scSNPbased cell assignments to donors.

c)

a)

b)


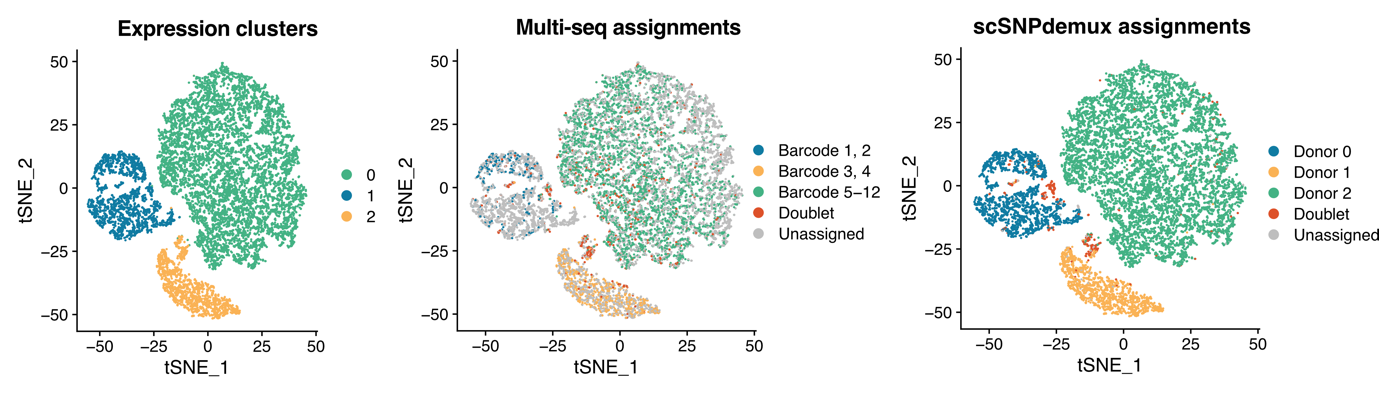


### Supplementary figure 8: UMI counts per cell across demultiplexing results

UMI counts per cell grouped by Multi-seq (a) and scSNPdemux (b) assignment.

a)

b)

## Supplementary information

### Supplementary Table 1

|  | **scSNPdemux** | | |  | **CellPlex** | | |  |
| --- | --- | --- | --- | --- | --- | --- | --- | --- |
|  | **assigned** | **unassigned** | **doublets** | **fraction of doublets [%}** | **assigned** | **unassigned** | **doublets** | **fraction of doublets [%}** |
| B-Cells | 3247 | 6 | 268 | 7,6% | 2060 | 1128 | 333 | 9,5% |
| T-Cells | 6355 | 23 | 621 | 8,9% | 4319 | 2138 | 542 | 7,7% |
| Others | 5050 | 15 | 541 | 9,7% | 4231 | 313 | 1062 | 18,9% |
| Tumour | 4302 | 19 | 624 | 12,6% | 3393 | 461 | 1091 | 22,1% |

Number of cells assigned und unassigned to a specific donor by scSNPdemux and CellPlex and doublets identified across different cell types.

### Supplementary Table 2 Assigments of the multiplexed Jurkat, HEK293 and HMEC cells from McGinnis et al. 2019

|  |  | Multiseq | | | | |
| --- | --- | --- | --- | --- | --- | --- |
|  | Cluster 0 | Barcode 1, 2 | Barcode 3, 4 | Barcode 5-12 | Doublet | Negative |
| scSNPdemux | donor0 | 0 | 0 | 0 | 0 | 2 |
|  | donor1 | 0 | 0 | 1 | 0 | 2 |
|  | donor2 | 0 | 1 | 3876 | 302 | 4541 |
|  | doublet | 0 | 0 | 9 | 2 | 52 |
|  | unassigned | 0 | 1 | 12 | 7 | 81 |
|  |  |  |  |  |  |  |
|  |  | Multiseq | | | | |
|  | Cluster 1 | Barcode 1, 2 | Barcode 3, 4 | Barcode 5-12 | Doublet | Negative |
| scSNPdemux | donor0 | 299 | 0 | 0 | 25 | 1652 |
|  | donor1 | 1 | 0 | 0 | 2 | 11 |
|  | donor2 | 0 | 0 | 0 | 1 | 1 |
|  | doublet | 4 | 0 | 9 | 49 | 92 |
|  | unassigned | 2 | 2 | 2 | 7 | 29 |
|  |  |  |  |  |  |  |
|  |  | Multiseq | | | | |
|  | Cluster 2 | Barcode 1, 2 | Barcode 3, 4 | Barcode 5-12 | Doublet | Negative |
| scSNPdemux | donor0 | 0 | 0 | 0 | 0 | 0 |
|  | donor1 | 0 | 634 | 1 | 41 | 752 |
|  | donor2 | 0 | 0 | 3 | 5 | 3 |
|  | doublet | 0 | 11 | 6 | 54 | 41 |
|  | unassigned | 0 | 15 | 1 | 8 | 13 |
|  |  |  |  |  |  |  |
|  | Overall number of cells | | 12665 |  |  |  |
|  | Not annotated by Multi-seq | | 7272 | 57,4% |  |  |
|  | Not annotated by scSNPdemux | | 180 | 1,4% |  |  |

### Supplementary Table 3 Overview of cells assigned by Multi-seq and scSNPdemux

Cells assigned by Multi-seq and scSNPdemux (McGinnis et al. 2019)

|  |  | Multiseq | | |
| --- | --- | --- | --- | --- |
| scSNPdemux | Cluster 0 | Barcode 1,2 | Barcode 3,4 | Barcode 5-12 |
|  | donor0 | 0 | 0 | 0 |
|  | donor1 | 0 | 0 | 1 |
|  | donor2 | 0 | 1 | 3876 |
|  |  |  |  |  |
|  |  | Multiseq | | |
| scSNPdemux | Cluster 1 | Barcode 1,2 | Barcode 3,4 | Barcode 5-12 |
|  | donor0 | 299 | 0 | 0 |
|  | donor1 | 1 | 0 | 0 |
|  | donor2 | 0 | 0 | 0 |
|  |  |  |  |  |
|  |  | Multiseq | | |
| scSNPdemux | Cluster 2 | Barcode 1,2 | Barcode 3,4 | Barcode 5-12 |
|  | donor0 | 0 | 0 | 0 |
|  | donor1 | 0 | 634 | 1 |
|  | donor2 | 0 | 0 | 3 |
|  |  |  |  |  |
|  | Contradicting assignments | | 7 |  |
|  | Assignments | | 4809 | 99,85% |

### Subclustering comparison between all identified cells and cells only identified by scSNPdemux

In order to determine if cells identified solely through scSNPdemux are based on noisy data or have biological significance, we conducted a separate subclustering analysis on the T-cell population. We compared all identified cells to those identified solely by scSNPdemux. Our clustering analysis indicates that T-cells identified solely by scSNPdemux clustered just as well as the subclustering analysis that included all demultiplexed and assigned cells (as shown in Supplementary Figure 5a). We also used established T-cell markers such as CD4, CD8, and CXCL13 to confirm the subclustering accurately reflected T-cell biology (as shown in Supplementary Figure 6). These findings support our argument that cells identified through scSNPdemux but not by CellPlex provide biologically meaningful information for the overall single cell analysis.
